# Sarracenia Purpurea as a Remedy for Small-Pox

**Published:** 1868-09

**Authors:** W. F. M’Nutt

**Affiliations:** Late U. S. N., Surgeon to S. F. Dispensary, etc.


### Sarracenia Purpurea as a Remedy for Small-Pox.

BY W. F. M'NUTT, M. D., M. R. C. S. E., L. R. C. P. E., ETC.
Late U. S. N., Surgeon to S. F. Dispensary, etc.

The inquiry on every hand at present seems to be: What is the best preventive of small-pox? And what is the best treatment if one does get victimized? Is there no royal road to cure? Can not the horrid disease be "stamped out?" Much has been said and written on the subject lately, and various are the theories propounded for the prevention, cure, and stamping out of the disease.

As for the treatment, perhaps no remedy has been so lionized by the popular journals of the city as the Sarracenia purpurea, purporting to be brought for the first time before the profession by Dr. F. W. Morris, of Nova Scotia, he having obtained it from "an old Indian woman."

I have been repeatedly called upon by gentlemen of this city, who knew that I had formerly prac'iced medicine in Nova Scotia, for my opinion of Sarracenia purpurea as a remedial agent in small-pox, and was strongly urged to give publicity to the facts in regard to its introduction to use by Dr. F. W. Morris. I considered it unnecessary to do so, however, and would not now occupy valuable space in your medical journal, had its use not been strongly recommended in the previous number by a member of the profession.

The story went in some of the popular journals that the Indians kept it in their camps, and found its use both a preventative and a cure, whereas it is a patent fact that no disease that ever followed the pale face to Nova Scotia proves so fatal to the Indian as the small-pox. When the small-pox makes its appearance in the neighborhood of the Indians, they never stop to try the virtues of Sarracenia purpurea, either as a prevention or cure, but immediately pull up their stakes and give the small-pox a wide birth. So much for the "old Indian woman."

At the time of the announcement in 1861, by Dr. F. W. Morris, of the specific effects of Sarracenia purpurea in small-pox, the disease was raging in the whole Province, but more especially in Halifax. The Medical Society at once appointed a committee for the purpose of thoroughly trying the remedy, and of investigating the cases that Dr. F. W. Morris had claimed to cure by the new medicine. In the meantime many of the members of the society commenced administering the drug to their patients. The committee in due time reported the result of their labors. They were unanimous in their opinion that Sarracenia purpurea is of no value as a remedial agent in the treatment of small-pox. The other members of the society also came to the same conclusion. The committee also found that the cases which Dr. F. W. Morris had reported as cured, were many of them badly marked, and that there was no evidence that any of them had been shortened or modified by Dr. F. W. Morris' treatment. My friend Dr. Davies, now of this city, was in Halifax at the time, and formed one of the committee. The consequence was, that Dr. F. W. Morris was expelled from the Medical Society for unprofessional conduct, and for the publication of false certificates of cure.

Dr. Morris then associated himself with a noted character who called himself Prof. Lane, and they two cried martyrdom, so that a few, out of sympathy for these professional martyrs, continued to take Sarracenia purpurea, as administered by Dr. Morris and Prof. Lane. Beyond that it ceased to be used in the Province. So much for Dr. F. W. Morris.

In Rainsford Island Small-pox Hospital, Boston harbor, in 1863 and 1864, Sarracenia purpurea was thoroughly tried under the superintendence of Dr. George E. Underwood. It was then condemned as useless in the treatment of small-pox. My friend, Dr. Powers, oculist, of this city, was physician at the hospital at that time.

Two species of this plant—Sarracenia flava and Sarracenia variolaris—have long been in use in South Carolina as a domestic remedy for dyspepsia. Dr. Porcher, in a communication to the Charleston *Medical Journal and Review*, calls these species a stomachic stimulant and laxative, and thinks that they also act as a stimulant to the brain and kidneys. For a full description of the Sarracenia purpurea, see a paper by Prof. Bentley in the *Pharmaceutical Journal*, for January, 1863.*—*California Med. Gazette.*

* We have heard, otherwise, sensible physicians speak confidently of the virtues of this drug in small-pox, and re-publish the above for their benefit. It seems wholly impossible that reasonable physicians should be imposed upon by such nonsense.—Ed.

